# Characterization of phospholipid profiles of egg yolks: Newly classified plasmalogens, distribution of polyunsaturated fatty acids, and the effects of dietary enrichment

**DOI:** 10.1016/j.fochx.2024.102105

**Published:** 2024-12-19

**Authors:** Le Cheng, Xinlei Yuan, Ming Zhang, Jianguo Dong, Yao Wu, Rang Wang, Yixuan Li, Lishui Chen, Bing Fang

**Affiliations:** a Key Laboratory of Precision Nutrition and Food Quality, Department of Nutrition and Health, China Agricultural University, Beijing 100083, China; b College of Food Science and Engineering, Tianjin University of Science and Technology, Tianjin 300457, China; c School of Food Science and Chemical Engineering, Beijing Technology and Business University, Beijing 100048, China; d Food Laboratory of Zhongyuan, Luohe 462300, Henan, China

**Keywords:** Egg yolk, Lactosylceramides, Gangliosides, Plasmalogen, Polyunsaturated fatty acids, Esterification position, Lipidomics

## Abstract

Egg yolk phospholipids are commercially valuable products that are beneficial to human health. Previous research on phospholipids in egg yolk mainly focuses on phosphatidyl choline (PC), phosphatidyl ethanolamine (PE), and fatty acid compositions, and neglects the esterification position and other bioactive phospholipids. This study found a total of 19 classes of phospholipids and 275 subclasses using lipidomics. The study firstly found that egg yolks were also rich in glucosylceramides, galactosylceramides, lactosylceramides, gangliosides, and plasmalogens with polyunsaturated fatty acids (PUFAs) at the high bioavailable *sn-2* position. Docosahexaenoic acid (DHA), eicosapentaenoic acid (EPA), α-Linolenic acid (ALA), and arachidonic acid (ARA) were esterified at *sn-1* position of PC and *sn-2* position of PE, phosphatidyl inositol (PI) and phosphatidic acid (PA). Microalgae feeding contributed to the deposition of PUFAs at *sn-2* position and increased the contents of plasmalogens. The results provided detail the phospholipid profiles of egg yolk to improve understanding of its nutrition.

## Highlights

• A total of 275 species in 19 phospholipids subclasses were found in egg yolk.
• Glycosphingolipids and plasmalogens were detected for the first time in egg yolk.
• Polyunsaturated fatty acid was esterified at *sn-1* site of phosphatidyl choline.
• Polyunsaturated fatty acid was found at *sn-2* site of phosphatidyl ethanolamine.
• Microalgae feeding enriched glycosphingolipids and phospholipids in egg yolk.

## 1 Introduction

Eggs are considered to be one of the most popular foods worldwide, provide most of the nutrients needed for human health, and are widely recommended by various healthy diets, such as the Mediterranean diet and Dietary Approaches to Stop Hypertension (Picklo et al., 2022). Egg yolk is an important component of eggs, accounting for about 30 % of the total mass. It is highly regarded for its rich nutrition, including phospholipids, proteins, vitamins, and minerals (Khalid, Chaudhary, et al., 2024; Khalid, Zahid, et al., 2024). Recently, the health effect of egg yolk phospholipids in human has received considerable attention, as they were found to be antioxidant (Sun et al., 2019), anti-inflammatory (Andersen, 2015), neuroprotective (Bao et al., 2020), and able to regulate lipid metabolism (Meng et al., 2024). Other studies reported that egg yolk phospholipid consumption benefitted cognitive performance and reduced the risk of Alzheimer's disease (Pan et al., 2024). Egg yolk phospholipids play an important role in these healthy benefits (Salahuddin et al., 2024).

Phospholipids account for only about 30 % of the total egg yolk lipids, however, and the phospholipid profile of egg yolk has not been fully clarified. Many studies have focused on the major phospholipids, such as phosphatidyl choline (PC), phosphatidyl ethanolamine (PE) and sphingomyelin (SM), while neglecting trace phospholipids components such as glucosylceramides (GlcCers), galactosylceramides (GalCers), lactosylceramides (LacCers), and gangliosides (GM3s), which have been reported to be beneficial for neural development (Neijat et al., 2020; Wang, Song, et al., 2023; Wang, Wang, et al., 2023). Furthermore, the site at which polyunsaturated fatty acids (PUFAs) are bound in the glycerol chain of phospholipids, such as the end *sn-1/3* or the middle *sn-2* site, largely influences the bioavailability and function of phospholipids *in vivo* (Murota, 2020). Whereas existing research has mainly measured the content of PUFAs but not the esterification sites of PUFAs in different subtypes of phospholipids. Recently, a new classification of phospholipids emerged according to the chemical bond at the fatty acid-binding position. Plasmalogens are a special type of phospholipid with a vinyl ether bond at the *sn-1* position and an ester bond at the *sn-2* position, and were reported to improve synaptic plasticity and delay brain aging (Xiong et al., 2024). Existing phospholipids studies have not reported the plasmalogen profile in egg yolks.

Adding nutrients to the diet of hens is an effective means of enriching the biological activity of chicken meat and egg yolks, which promotes the application of egg yolk products and has become a trend in the food industry (Ciptaan et al., 2024; Liaqat et al., 2023; Marcet et al., 2022; Shamsi et al., 2023; Srifani et al., 2024). Microalgae, as an important source of PUFAs, has been widely added to animal feeds to enhance the nutritional value of food products (Khalid, Chaudhary, et al., 2024; Khalid, Zahid, et al., 2024). For example, supplementing the diet of hens with 2 % *Schizochytrium limacinum* and 23 % *Nannochloropsis oceanica* was reported to increase the EPA and DHA content of eggs (Ao et al., 2015; Manor et al., 2019). Microalgae are also rich in sphingolipids, such as Cers, GM3s, and plasmalogens (Li et al., 2017). However, the effects of dietary microalgae supplements on phospholipid profiles and the nutritional value of egg yolk remain unclear.

Lipidomics has been widely used in food science research to study lipid components from various biological sources, and it can support extensive and large-scale investigations on lipids. (Tan et al., 2023). Numerous studies have used lipidomics to investigate chemical changes in the phospholipid profile of various food: Liu et al. (2023) used lipidomics to identify changes in egg yolk lipids during storage, and found that major lipids were involved in glycerophospholipid and glyceride metabolism. Other studies used lipidomics technology to determine the position of PUFAs in *Scylla paramamosai* (mud crab) (Wang et al., 2024) and the content of plasmalogens in milk (Liu, Liu, Niu, et al., 2024; Liu, Liu, Zhao, et al., 2024), providing a basis for using lipidomics to elucidate the lipid profile of egg yolks, including the PUFAs positions and plasmalogens enriched by feed supplements.

Therefore, in this study, we investigated the phospholipid profiles of egg yolks based on representative samples from Chinese laying hens fed different feeds. The effects of microalgae diets and the correlation and potential metabolic pathways of significantly different phospholipids (SDPs) were also compared. This study aimed to provide data to accurately describe the nutritional lipid components of egg yolk and provide a theoretical basis for analyzing the health effects of eggs. Furthermore, diet-induced phospholipid changes may uncover unidentified mechanisms by which lipid profiles are changed in eggs through nutritional supplements.

## 2 Materials and methods

### 2.1 Sample preparation

Eggs were collected with detailed information (Beijing, China). The eggs were produced from Hailan Brown laying hens, which were fed a basic diet formula (control diet) based on grains. An experimental diet formula also based on grains was supplemented with 3 % microalgae (*Aurantiochytrium limacinum*). The eggs from the two groups were manually cracked open and the egg yolks were collected by separating them from the egg whites and piercing the yolk membrane. Ten egg yolks were obtained from each group, then mixed thoroughly, rapidly frozen in liquid nitrogen, and stored at −80 ± 1.0 °C for further testing.

### 2.2 Extraction of phospholipids

A modified version of the method reported by Xu et al. (2022) was used to extract lipids from about 30 mg of the frozen samples. Briefly, 750 μL of a 3:6:1 (*v*/v/v) chloroform:methanol: MilliQ H_2_O solution was used to homogenize the egg yolks. After a 1 h incubation period at 4 °C and 1500 rpm, 350 μL of deionized water and 250 μL of chloroform were added. After centrifuging the samples at 3000*g*, the lipid-containing lower organic phase was removed and placed in a clean tube. The leftover lipids in the aqueous phase were extracted once by adding 450 μL of chloroform. The extracts were then combined into one tube and dried using a SpeedVac vacuum concentrator (Thermo Scientific, Waltham, MA, USA) under OH mode.

### 2.3 Quantitative analysis of the egg yolk lipidomics

An Agilent 1290 UPLC system (Santa Clara, USA) coupled with a triple quadrupole/ion trap mass spectrometer (Framingham, USA) was used, following a method reported by Lam et al. (2021). Using a TUP-HB silica column (i.d. 150 × 2.1 mm, 3 μm) and mobile phases A (ammonium hydroxide: methanol: chloroform = 0.5:10:89.5) and B (ammonium hydroxide: water: methanol: chloroform = 0.5:5.5: 39: 55), the various polar lipid classes were isolated by normal phase-HPLC. The multiple reaction monitoring transitions were used to compare different polar lipids. By spiking with internal standards as a reference, the content of the different lipid species was measured. The internal standards information is shown in Table S1. The phospholipid levels are shown in μmol/g.

### 2.4 Statistical analysis

The egg yolk phospholipid analysis was conducted three times. Analysis of variance (ANOVA) was used to analyze the results, and the mean ± SD was used to express the results. SPSS software (version 20.0, IBM Corp., USA) was used for statistical analyses. *p* < 0.05 was considered statistically significant.

## 3 Results and discussion

### 3.1 Phospholipid profiles of egg yolk

**Fig. 1:**
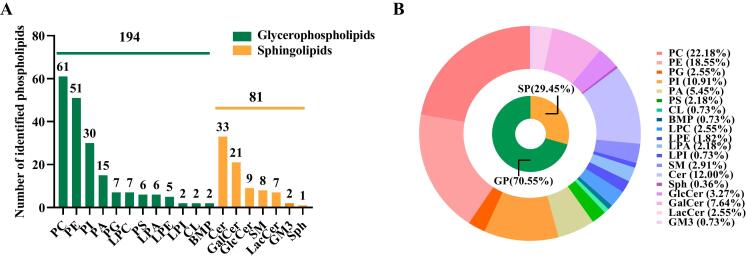
Number of phospholipid species identified in 2 classes and 19 phospholipid subclasses (A). Percentage of each phospholipid subclass and class (B).

A total of 275 phospholipids were identified from yolks in the control group (CY) and from the microalgae-supplemented group (MY) (Fig. 1A). The phospholipids were grouped into two categories: glycerophospholipids (GP; *n* = 194, 70.55 %) and sphingolipids (SP; *n* = 81, 29.45 %). The two categories were then further classified to better analyze the metabolic pathways of phospholipid molecular transformations in the egg yolks. Fig. 1A, B shows the 12 subclasses in the GP category, which included 61 PCs (22.18 %), 51 PEs (18.55 %), 30 PIs (10.91 %), 15 PAs (5.45 %), 7 phosphatidyl glycerols (PGs, 2.55 %), 6 phosphatidyl serines (PSs, 2.18 %), 7 lysophosphatidyl cholines (LPCs, 2.55 %), 6 lysophosphatidic acids (LPAs, 2.18 %), 5 lysophosphatidyl ethanolamines (LPEs, 1.82 %), 2 lysophosphatidyl inositols (LPIs, 0.73 %) 2 cardiolipins (CLs, 0.73 %), and 2 bis (monoacylglycero)phosphates (BMPs, 0.73 %). The 7 subclasses in the SP category included 33 ceramides (Cers, 12.00 %), 21 GalCers (7.64 %), 9 GlcCers (3.27 %), 8 SMs (2.91 %), 7 LacCers (2.55 %), 2 GM3s (0.73 %), and 1 sphingosine (Sph, 0.36 %). Clearly, PC, PE, Cer, and PI comprised the majority of the egg yolk phospholipids. The main phospholipid species identified here were consistent with the observations of Liu et al. (2023), indicating the importance of PC, PE, Cer, and PI in the diversity of egg yolk phospholipids. The quantity of phospholipid species identified in this investigation was higher than that of a previous study (Hu et al., 2024), demonstrating that the phospholipid profile of egg yolks could be expanded. This study found for the first time changes in GalCer, GlcCer, LacCer and GM3 contents in egg yolk phospholipids. The supplementation of phospholipid profiles in this study can provide ideas for developing nutritional fortifiers for egg production in the future.

**Fig. 2:**
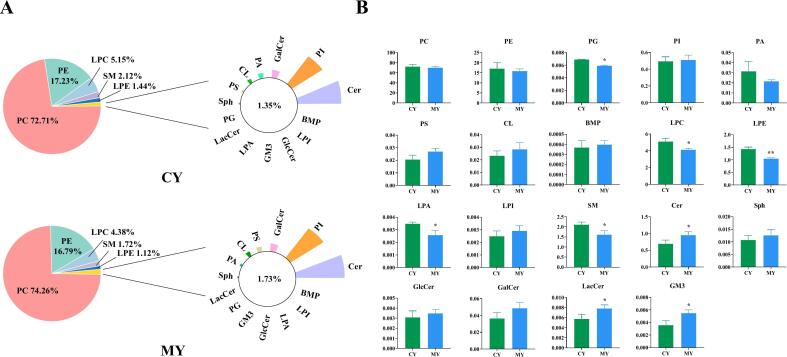
Percentage of the content of each phospholipid subclass (A). Comparison of phospholipid subclass contents (B).

The total combined content of the identified phospholipid species was used to compute the relative content of each phospholipid. Fig. 2A and Table S2 show that PC had the largest percentage in the CY (72.71 %) and MY (74.26 %) groups, followed by PE (CY, 17.23 %; MY, 16.79 %), LPC (CY, 5.15 %; MY, 4.38 %), SM (CY, 2.12 %; MY, 1.72 %), LPE (CY, 1.44 %; MY 1.12 %), and Cer (CY, 0.70 %; MY, 1.01 %). The percentage of PC in egg yolks was the largest, almost 70 % in all samples; however, the distribution of the other phospholipids differed according to whether the egg yolks were from chickens fed the supplemented or control diet. Ali et al. (2017) found similar results for phospholipids from duck, hen and quail egg yolks in which PC formed the highest proportion of phospholipids followed by PE. Fig. 2B shows a further comparison of the distribution of 19 lipid subclasses in the two groups of egg yolks. The results showed that the content of PC was 72.19 ± 3.88 μmol/g in the CY group and 70.02 ± 1.99 mg/g in the MY group, and the content of PE was 17.11 ± 2.35 mg/g in the CY group and 15.83 ± 0.90 mg/g in the MY group. The content of LPC, SM, LPE, PG, and LPA decreased more significantly in the MY group than in the CY group, whereas the content of Cer, LacCer, and GM3 increased more significantly in the MY group (*p* < 0.05). Cer is a type of sphingomyelin with a sphingosine base that binds fatty acids through amide bonds. Cer is intimately linked to the early development and immunological control of the brain and nervous system and performs various regulatory functions, including cell signal transduction, membrane transport, and cell apoptosis (Liu, Liu, Niu, et al., 2024; Liu, Liu, Zhao, et al., 2024). The results here indicated that egg yolks were enriched with ceramides, and microalgae feeding significantly enhanced the concentration of ceramides, thus emphasizing the importance of supplementing ceramides in egg yolks to maintain their nutritional quality. The egg yolks also contained LacCer, and microalgae feeding increased its content. LacCer, a glycosphingolipid, is a conjugated lipid composed of ceramides, lactose, and glucose chains, and plays important roles in cellular recognition and communication, particularly in the nervous system (Li et al., 2021). Another type of glycosphingolipid is GM3, which comprises sialic acid and oligosaccharide residues joined by many bonds. GM3 is typically found in breast milk and is essential for the development of the infant brain and intestinal immunity (Yang et al., 2024). The results indicated that the content of GM3 in egg yolks from hens with microalgae-supplemented increased, thus indicating the potential of microalgae as a dietary supplement for pregnant women and young children. The results also showed that the content of SM was 2.11 ± 0.11 μmol/g in the CY group, and 1.62 ± 0.15 μmol/g in the MY group, which may be explained by the action of sphingophospholipase which degrades sphingophospholipids to produce ceramides and phosphatidylcholine (Yi et al., 2023).

### 3.2 Distribution of esterified PUFAs in phospholipids

**Fig. 3:**
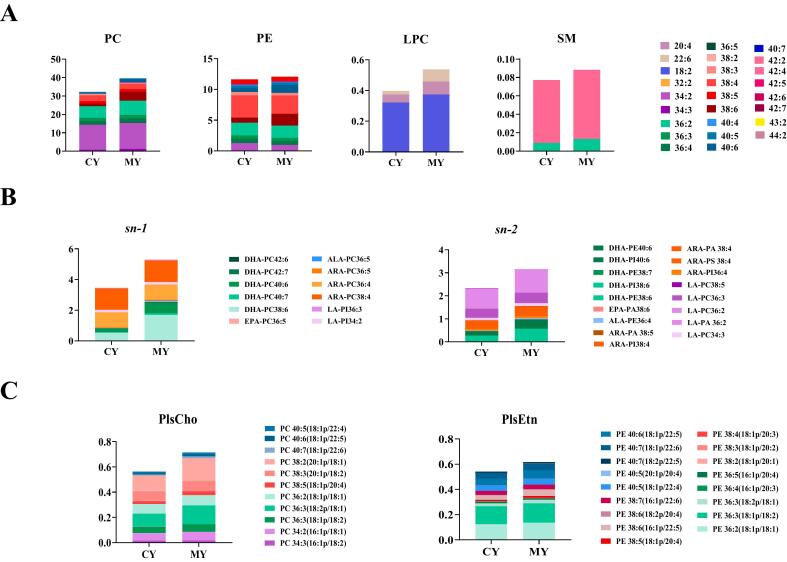
PUFAs of phospholipids (A). Esterification site of DHA, EPA, and ALA (B). Plasmalogen contents (C).

PUFAs refer to fatty acids with two or more double bonds, which have many health benefits in neural development and the prevention of cardiovascular diseases, especially long-chain (>C18) PUFAs. Compared to free PUFAs, PUFAs esterified in phospholipids are more easily absorbed (Sugasini et al., 2019). However, the current research only focuses on the free content of PUFAs and has not delved into the PUFAs within phospholipids in detail. Therefore, it is very important to study details of the content of PUFAs in egg yolk phospholipids. The PUFAs compositions of PC, PE, LPC, and SM, the top four phospholipids with the highest content in the CY and MY groups, were compared (Fig. 3A and Table S3). The PUFAs composition of each phospholipid in the CY and MY groups differed, and the PUFAs species and contents in the PC and PE were more diverse than those in the LPC and SM. The PCs exhibited higher contents of PUFAs, including PC34:2, PC36:2, and PC38:4, which were significantly higher in the MY group than in the CY group. The PE species contained PUFAs such as PE38:4, PE38:6, PE38:5, PE38:6, and PE40:6 in the CY group was significantly lower than in the MY group (*p* < 0.05). LPC and SM also showed similar results, with the contents of LPC20:4, LPC22:6, LPC18:2, SM36:2, and SM42:2, significantly higher in the MY group than in the CY group. The results confirmed that microalgae feeding increased specific types of long-chain PUFAs in phospholipids in egg yolks, which were more easily digested and absorbed by the human body, thereby improving the nutritional characteristics of eggs.

The esterification positions of DHA, EPA, ALA, ARA, and linoleic acid (LA) in egg yolk phospholipids were thoroughly characterized. Fig. 3B and Table S4 show the distribution of DHA, EPA, and ALA at the *sn-1* and *sn-2* sites of phospholipids in the two groups of egg yolk samples. In the egg yolk phospholipids, DHA, EPA, and ALA were mainly distributed at the *sn-1* position in PC, and DHA-PC had the highest content. The top three DHA-PCs in the CY group were DHA-PC38:6, DHA-PC40:6, and DHA-PC40:7, and the content of each in the MY group were 3.19, 3.08, and 2.67 times that in the CY group, respectively. This indicated that microalgae feeding significantly increased the DHA content at the *sn-1* site of PC. Compared with the control group, microalgae feeding significantly increased the content of EPA-PC36:5 at the *sn-1* site from 0.0056 ± 0.0007 μmol/g to 0.0431 ± 0.0037 μmol/g, and the content of ALA-PC36:5 at the *sn-1* site from 0.0326 ± 0.0054 μmol/g to 0.0611 ± 0.0070 μmol/g. The number of lipid types and the content of EPA measured in the sample were lower than that of DHA, which may be related to the preferential conversion of EPA to DHA in eggs (Neijat et al., 2016). Wang, Song, et al. (2023) and Wang, Wang, et al. (2023) also found a similar phenomenon in egg yolks rich in DHA and in ordinary samples. DHA was concentrated in PC, and the content of EPA in the phospholipids was much lower than that of DHA. The content of ALA-PC36:5 at the *sn-1* site in the MY group was 1.87 times higher than that in the CY group. This was attributed to the mutual conversion between DHA and ALA in microalgae during the digestion and absorption processes. ARA was mainly distributed at the *sn-1* position in PC and LA was mainly distributed at the *sn-1* position in PI. DHA was mainly bound to the *sn-2* site of PE, as PE38:6 (16:0/22:6), PE40:6 (18:0/22:6), and PE38:7 (16:1/22:6). The content of each of these species in the MY group was 2.08, 2.09, and 2.08 times that in the CY group, respectively. DHA at the *sn-2* position of the phospholipids was found to be more stable and well absorbed by the intestinal mucosa, with high bioavailability (Wang et al., 2024). These results indicated that DHA-PE was more stable than DHA-PC in egg yolks, and that microalgae feeding increased the DHA-PE content, which was more conducive to improving the nutritional value of eggs. The contents of EPA-PA38:6 and ALA-PE36:4 in the MY group were also higher than those in the CY group. ARA also showed similar results and was found at the *sn-2* site of PA, PI, and PS. LA was found at the *sn-2* site of PC and PA. These results indicated that the distribution of DHA, EPA, ALA and ARA in egg yolk favored the *sn-1* position in PC and the *sn-2* position in PE, PI, and PA. Supplementation with microalgae, with a higher content of long-chain unsaturated fatty acids, promoted this trend. Previous studies showed that unsaturated fatty acid chain length affected the absorption process of dietary phospholipids from the intestinal cavity to the intestinal cells (Xu et al., 2021). In contrast to short-chain and medium-chain unsaturated fatty acids, the long-chain unsaturated fatty acids preferentially attached to fatty acid binding proteins (Neijat et al., 2016).

### 3.3 Plasmalogen profiles of egg yolk

The molecular structure of plasmalogens comprise a phosphate headgroup at positions *sn-1*, *sn-2*, and *sn-3* of the glycerol backbone, a vinyl-ether-linked chain, and an ester-linked fatty acyl chain. Choline plasmalogens (PlsChos) and ethanolamine plasmalogens (PlsEtns) are the two primary types of plasmalogens according to headgroup. As shown in Fig. 3C and Table. S5, the contents of PlsCho and PlsEtn were 0.72 ± 0.09 μmol/g and 0.62 ± 0.05 μmol/g in the MY group, which were significantly higher than in the CY group of 0.56 ± 0.03 μmol/g and 0.54 ± 0.01 μmol/g, respectively (*p* < 0.05). In addition, we found that plasmalogens were a reservoir of PUFAs, and that microalgae feeding increased the content of PUFAs in plasmalogens. The three PlsChos with the highest content were PC38:2 (20:1p/18:1), and PC36:3 (18:2p/18:1), and PC38:3 (20:1p/18:2), and the content of these in the MY group were 1.5, 1.5, and 1.1 times higher than in the CY group, respectively. The top three PlsEtn were PE36:3 (18:1p/18:2), PE36:2 (18:1p/18:1), and PE40:6 (18:1p/22:5), and the content of each of these in the MY group was 1.1, 1.2, and 1.4 times higher than in the CY group, respectively. DHA was more easily esterified at the *sn-2* position of PlsCho and PlsEtn, which was consistent with the results of plasmalogens in skeletal muscle of moose (Pham et al., 2021). This result proposes for the first time that egg yolk contained plasmalogens, and that microalgae supplementation can increase the plasmalogen content and enrich PUFAs at their *sn-2* position, thereby enhancing the health benefits of egg yolks in brain development and neuronal protection.

### 3.4 Effects of dietary enrichment

**Fig. 4:**
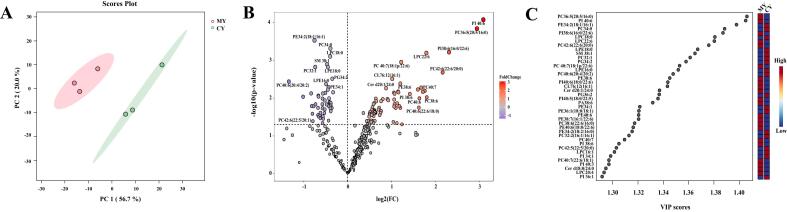
Plot of PCA scores (A). Volcano plot (B). VIP scores of phospholipid species (C).

To further explore the overall distribution of egg phospholipids and screen phospholipid metabolites from hens fed microalgae, the phospholipid identification data were used to create a multilevel statistical analysis model. These methods comprised multivariate statistical analysis (unsupervised PCA) and univariate statistical analysis (*t*-test and fold change [FC]). Fig. 4A shows PCA score plot. The first principal component (PC1) accounted for 56.7 % of the dataset variance, while the second principal component (PC2) accounted for 20.0 % of the data variance. PC1 and PC2 thus described a variance of 76.7 %, indicating that the phospholipid molecules in the MY group were significantly separated from those in the CY group. Furthermore, the good reproducibility of each group of parallel samples reflected the representativeness of the selected samples and the stability of the measurement process. A volcano plot was used to identify the downregulated (FC < 1.0 and *p* < 0.05) and upregulated (FC > 1.0 and *p* < 0.05) lipids (Fig. 4B). Each lipid species is represented by a point on the volcano map. The logarithm (base 2) of the FC is represented by the horizontal axis, and the common logarithm of the *p* value is represented by the vertical axis. The greater the absolute value of the abscissa, the greater the FC of expression between the two samples; the more significant the differential expression and the more dependable the screened species, the greater the vertical axis value. The downregulated phospholipid molecules are shown as blue dots, the upregulated phospholipid molecules are shown as red dots, and the phospholipid molecules with insignificant differences are shown as gray dots. Fig. 4B shows that, compared with the CY group, the MY group had 85 significantly upregulated and 51 significantly downregulated phospholipids. Potential differential phospholipid molecules were chosen for additional screening based on variable impact projection (VIP) > 1.3, which was determined by comparing the lipid data and computing VIP values for each lipid (Fig. 4C).

**Fig. 5:**
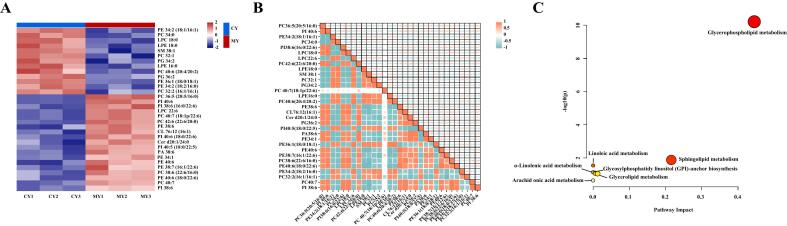
Heat map analysis of 31 SDPs between the CY and MY groups (A). Analysis of the correlation of SDPs (B). Green dots represent negative correlations and red dots represent positive correlations. Metabolomic view of important phospholipid biosynthesis pathways (C). (For interpretation of the references to color in this figure legend, the reader is referred to the web version of this article.)

The heatmap (Fig. 5A) shows that 31 SDPs were identified between the CY group and the MY group, including 9 PCs, 8 PEs, 4 PIs, 2 LPCs, 2 PGs, 2 LPEs, 1 SM, 1 CL, 1 Cer, and 1 PA (Table S6). Of the 9 PCs, the total carbon number ranged from 32 to 42, and mainly comprised long-chain polyunsaturated fatty acids, with double bond numbers distributed between zero and seven. The content of phospholipids containing saturated fatty acids in the MY group was significantly lower than that in the CY group, and included PE34:0, LPC18:0, LPE18:0 and LPE16:0. However, phospholipids containing polyunsaturated fatty acids, especially those rich in DHA and EPA in the MY group, were significantly higher than in the CY group, including PC36:5 (20:5/16:0), PI38:6 (16:0/22:6), PC42:6 (22:6/20:0), PI40:6 (18:0/22:6), PE38:7 (16:1/22:6), PC38:6 (22:6/16:0) and PE40:6 (18:0/22:6), which may be due to the abundance of DHA and EPA in the microalgae. Perdana et al. (2021) showed that the content of EPA in microalgae accounted for about 0 %–27.1 % of total fatty acids, and DHA accounted for about 0 %–42.9 %. Among the significantly different lipids, although the levels of EPA-PL and DHA-PL both increased in the MY group, DHA was more commonly found at the *sn-2* position, which was more conducive to improving the stability of the egg yolk phospholipid and supporting the brain function. The content of Cer d20:1/24:0 in the MY group increased compared with that in the CY group, indicating that microalgae contributed to the presence of ceramides in egg yolks, thus promoting brain neurodevelopment and cognitive ability in infants, and delaying the aging process in the elderly brain. The content of PC40:7 (18:1p/22:6) in the MY group was also significantly higher than that in the CY group, indicating that microalgae feeding increased the content of PlsCho and enriched DHA at the *sn-2* position, thereby improving the stability and bioavailability of DHA-PlsCho.

Based on the similarity of lipid structures and physiological traits found in this study, Pearson correlation analyses were performed to determine the association between the significantly different phospholipids found between the CY and MY groups. Correlation networks of 31 significantly different phospholipids were constructed, which confirmed the interactions of these phospholipids in egg yolks from hens fed microalgae (Fig. 5B). The findings demonstrated a significant degree of connections among the majority of phospholipid types and greater interactions among the same types of phospholipids. In the MY group, PE38:6 showed a negative correlation with LPE18:0 and LPE16:0. This is possibly because the LPE in the yolk of eggs from hens fed microalgae was more easily converted to PE under the catalysis of lysophosphatidylethanolamine acyltransferases (LPEAT), thus improving the nutritional value of the eggs (Manor et al., 2019). Cer d20:1/24:0 was positively correlated with PC36:5 (20:5/16:0) and PC42:6 (22:6/20:0). This indicated a close relationship between PC and Cer, which jointly have a role in cellular recognition and communication, and are particularly important in the development of the nervous system. Wang, Song, et al. (2023) and Wang, Wang, et al. (2023) have also found similar results in mature breast milk and ewe milk, with a positive correlation between PC (16:0/18:2) in the breast milk and Cer (d18:1/20:0) in the ewe milk. Cer d20:1/24:0, PC36:5 (20:5/16:0), and PC42:6 (22:6/20:0) were negatively correlated with SM38:1. This may be due to the conversion of sphingolipids into Cer and PC in egg yolks from hens fed microalgae. However, there was a weak correlation between PC40:7 (18:1p/22:6) and other phospholipids. This indicated that PC40:7 (18:1p/22:6) generally had a strong correlation with plasmalogens with the same structure (a vinyl ether bond at the *sn-1* position), but had a weak correlation with glycerophospholipids with different structures (Song et al., 2024).

To further explore the effects of the types and concentrations of phospholipids on metabolic pathways in the CY and MY groups, quantitative data corresponding to 31 SDPs between the CY and MY groups were imported into MetaboAnalyst 5.0 (www.metaboanalyst.ca/MetaboAnalyst/home.xhtml) to identify the most important and relevant lipid metabolic pathways. The pathway impact value and *p*-value were calculated using pathway topological analysis (Fig. 5C and Table. S7). Each bubble represents a metabolic pathway; the higher the horizontal coordinate value, the larger the bubble, indicating a more significant enrichment level. The larger the vertical coordinate value, the darker the bubble color, indicating a lower *p*-value. The seven lipid metabolic pathways with the highest correlations were identified: glycerophospholipid metabolism, sphingolipid metabolism, linoleic acid metabolism, α-linolenic acid metabolism, glycosylphosphatidylinositol (GPI)-anchor biosynthesis, glycerolipid metabolism, and arachidonic acid metabolism. The most significant lipid metabolic pathways were related to glycerophospholipid metabolism, which plays an important role in regulating cell function, and sphingolipid metabolism, which supports cell growth and signal transduction. In the MY group, PC (C00157), PE (C00350), PA (C00416), LPC (C04230), LPE (C04438), PG (C00344), and CL (C05980) were involved in glycerophospholipid metabolism (Fig. S1A), while SM (C00550) and Cer (C00195) were involved in sphingolipid metabolism (Fig. S1B). PC and PE were involved in glycerophospholipid metabolism and also enriched in many other metabolic pathways: glycosylphosphatidylinositol (GPI)-anchored biosynthesis, linoleic acid metabolism, α-linolenic acid metabolism, glycerolipid metabolism and arachidonic acid metabolism.

## 4 Conclusion

Overall, 275 phospholipids belonging to 19 phospholipid subclasses were identified in egg yolks. The content of PC was the highest, followed by PE. DHA, EPA, ALA, and ARA were esterified at the *sn-1* position of PC and at the *sn-2* position of PE, PI and PA. LA was esterified at the sn-1 position of PI and at the sn-2 position of PC. This result showed for the first time that egg yolk was rich in GlcCers, GalCers, LacCers, and GM3s, and also in plasmalogens with PUFAs at the sn-2 position. In addition, microalgae feeding increased the content of phospholipids and plasmalogens. Compared with the CY group, a total of 31 differentially metabolized lipids were identified in the MY group, which can serve as biomarkers for evaluating egg yolk quality. These were mainly enriched in the glycerophospholipid and sphingolipid metabolic pathways. These findings supplement the current dataset of egg yolk phospholipids and deepen the understanding of the mechanisms by which microalgae feeding facilitates changes in egg yolk phospholipids. Future studies will use larger sample sizes to confirm the usefulness of lipid biomarkers for elucidating the lipidomics differences between nutritionally fortified eggs and regular eggs. The absorption of plasmalogens and PUFAs in the human intestine also requires further exploration.

The following are the supplementary data related to this article.

Supplementary Figure S1 Metabolic pathways of significantly different phospholipids between the CY and MY groups. Supplementary Figure S1

Supplementary Table S1 Internal standard information. Supplementary Table S1

Supplementary Table S2 Content and relative abundance of phospholipid subclasses. Supplementary Table S2

Supplementary Table S3 Combinations of PUFAs of the phospholipids. Supplementary Table S3

Supplementary Table S4 Phospholipids containing PUFAs. Supplementary Table S4

Supplementary Table S5 Content of plasmalogens. Supplementary Table S5

Supplementary Table S6 Identification of 31 SDPs between CY and MY groups. Supplementary Table S6

Supplementary Table S7 Metabolic pathways identified from SDPs. Supplementary Table S7

## CRediT authorship contribution statement

**Le Cheng:** Writing – original draft, Software, Investigation, Formal analysis, Data curation, Conceptualization. **Xinlei Yuan:** Investigation, Formal analysis, Data curation. **Ming Zhang:** Writing – review & editing, Conceptualization. **Jianguo Dong:** Data curation. **Yao Wu:** Data curation. **Rang Wang:** Resources. **Yixuan Li:** Resources. **Lishui Chen:** Resources. **Bing Fang:** Writing – review & editing, Investigation, Funding acquisition, Conceptualization.

## Declaration of competing interest

The authors declare that they have no known competing financial interests or personal relationships that could have appeared to influence the work reported in this paper.

## Data Availability

Data will be made available on request.
