# Supplementary figures and images for: Characterization of phospholipid profiles of egg yolks: Newly classified plasmalogens, distribution of polyunsaturated fatty acids, and the effects of dietary enrichment
Source: Food Chem X. 2024 Dec 19;25:102105. doi: 10.1016/j.fochx.2024.102105 (PMC11732495; doi:10.1016/j.fochx.2024.102105)

**A**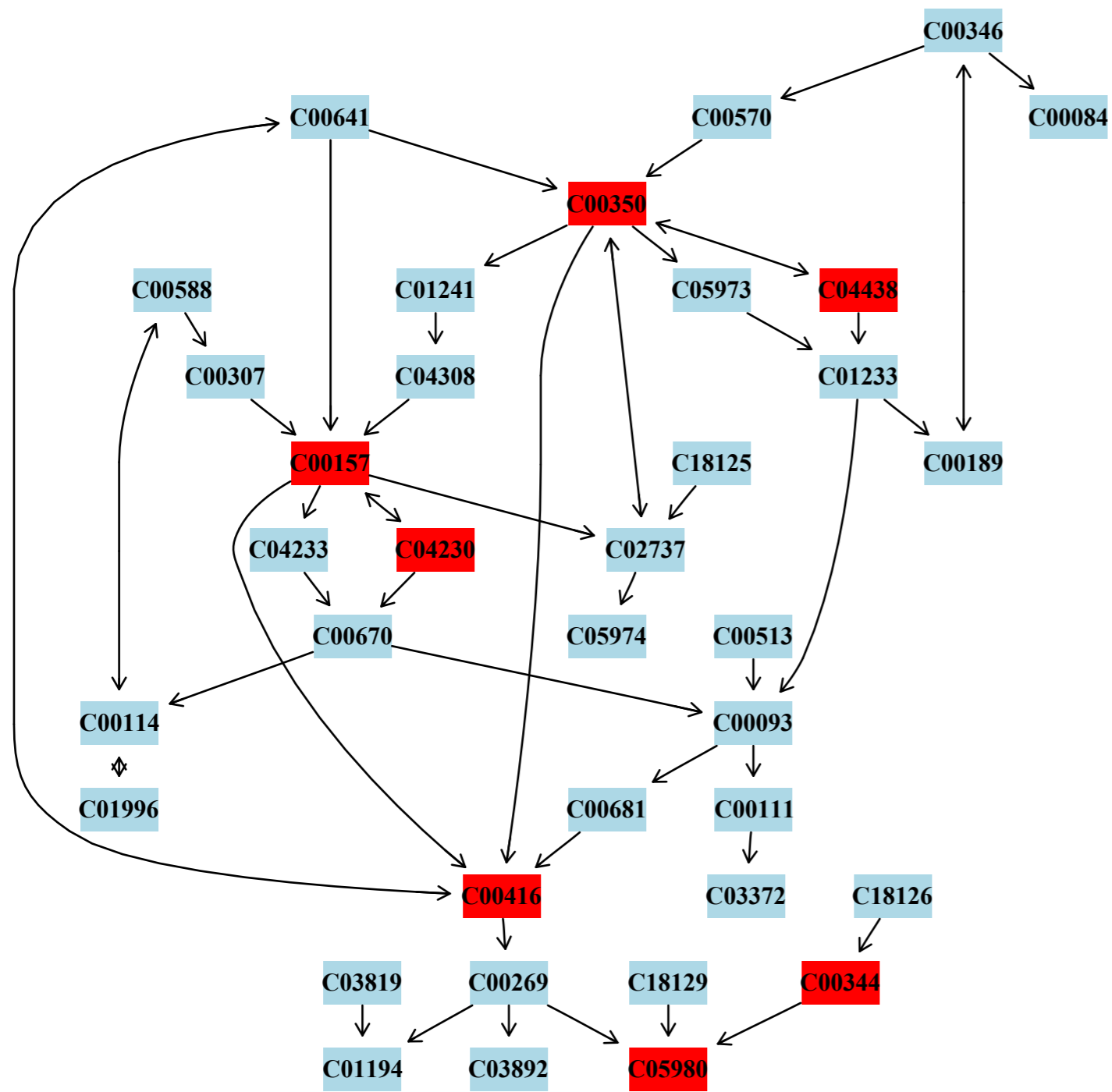**B**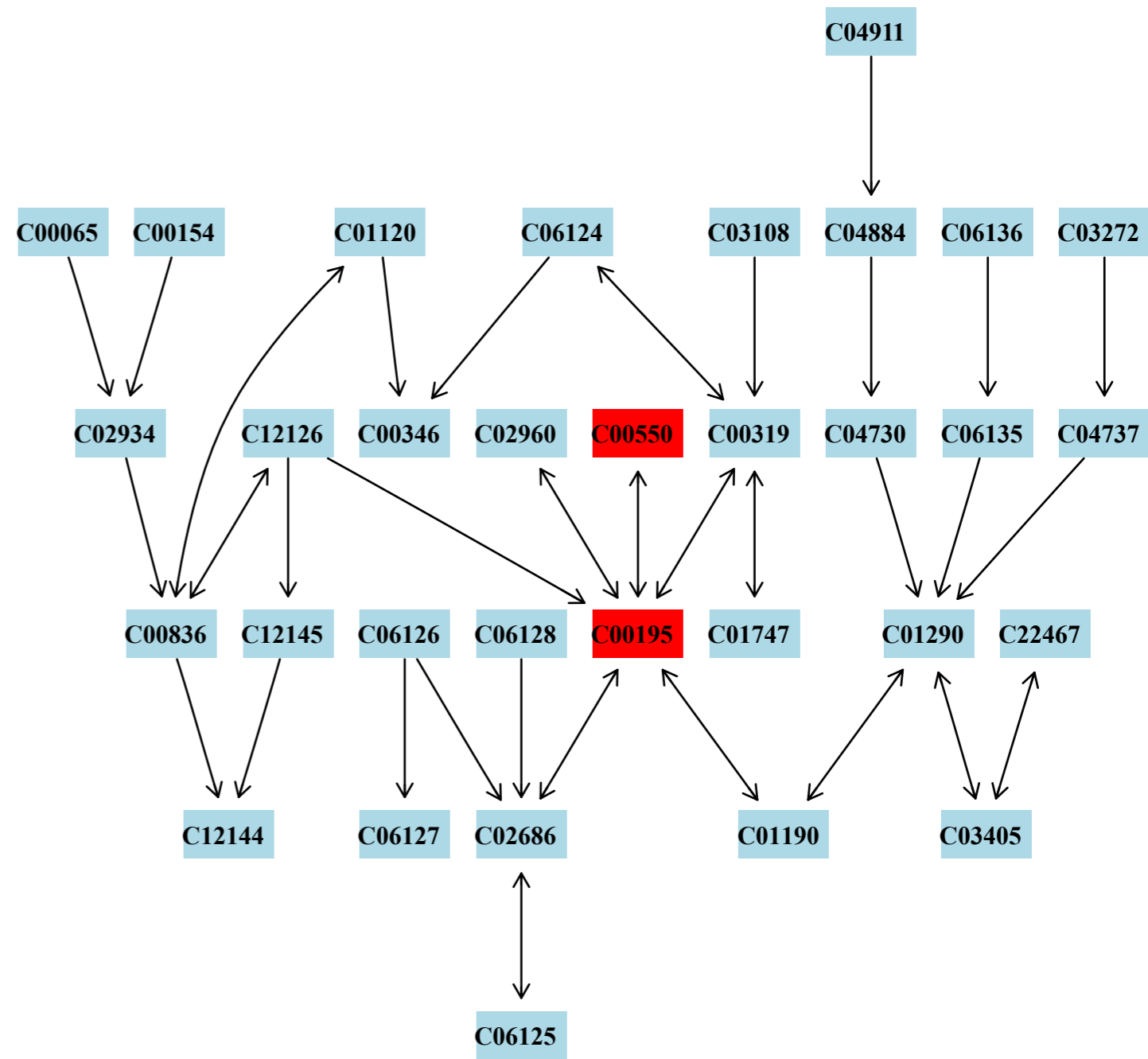

Supplement: Supplementary Figure S1 — Metabolic pathways of significantly different phospholipids between the CY and MY groups. [file mmc1.pdf]
